# Advances in Miniaturized Computational Spectrometers

**DOI:** 10.1002/advs.202404448

**Published:** 2024-10-30

**Authors:** Qian Xue, Yang Yang, Wenkai Ma, Hanqiu Zhang, Daoli Zhang, Xinzheng Lan, Liang Gao, Jianbing Zhang, Jiang Tang

**Affiliations:** ^1^ School of Integrated Circuits Huazhong University of Science and Technology (HUST) Wuhan 430074 P. R. China; ^2^ School of Optical and Electronic Information Huazhong University of Science and Technology (HUST) Wuhan 430074 P. R. China; ^3^ Wuhan National Laboratory for Optoelectronics (WNLO) Huazhong University of Science and Technology (HUST) Wuhan 430074 P. R. China; ^4^ Wenzhou Advanced Manufacturing Technology Research Institute Huazhong University of Science and Technology Wenzhou 325035 P. R. China; ^5^ Optics Valley Laboratory 1037 Luoyu Road Wuhan 430074 P. R. China; ^6^ Shenzhen Huazhong University of Science and Technology Research Institute Shenzhen Guangdong 518057 P. R. China

**Keywords:** compressive sensings, miniaturized computational spectrometers, reconstruction algorithms, spectral encoding

## Abstract

Miniaturized computational spectrometers have emerged as a promising strategy for miniaturized spectrometers, which breaks the compromise between footprint and performance in traditional miniaturized spectrometers by introducing computational resources. They have attracted widespread attention and a variety of materials, optical structures, and photodetectors are adopted to fabricate computational spectrometers with the cooperation of reconstruction algorithms. Here, a comprehensive review of miniaturized computational spectrometers, focusing on two crucial components: spectral encoding and reconstruction algorithms are provided. Principles, features, and recent progress of spectral encoding strategies are summarized in detail, including space‐modulated, time‐modulated, and light‐source spectral encoding. The reconstruction algorithms are classified into traditional and deep learning algorithms, and they are carefully analyzed based on the mathematical models required for spectral reconstruction. Drawing from the analysis of the two components, cooperations between them are considered, figures of merits for miniaturized computational spectrometers are highlighted, optimization strategies for improving their performance are outlined, and considerations in operating these systems are provided. The application of miniaturized computational spectrometers to achieve hyperspectral imaging is also discussed. Finally, the insights into the potential future applications and developments of computational spectrometers are provided.

## Graphical abstract

This review presents a comprehensive overview of miniaturized computational spectrometers, focusing on spectral encoding strategies and reconstruction algorithms. The analysis includes recent advances, optimization strategies, performance metrics, and considerations for practical applications. Additionally, the application of these spectrometers for hyperspectral imaging and insights into future developments are discussed, highlighting their potential for various fields.

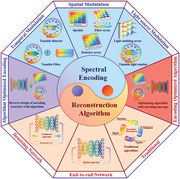

## 1 Introduction

Spectral sensing technology stands at the forefront of methodologies for delving into the complexity of the material field and examining the composition of various substances. Instruments crafted for this endeavor encompass wavelength sensors,^[^ ^1^, ^2^ ^]^ multispectral cameras,^[^ ^3^, ^4^ ^]^ and, notably, spectrometers.^[^ ^5^ ^]^ Spectrometers, in particular, excel in delineating the relationship between photoresponse and wavelength, offering merits such as improvement in analytical accuracy and the capability for non‐intrusive detection. Their applications span various disciplines, from astronomy^[^ ^6^, ^7^, ^8^ ^]^ and biomedical sensing^[^ ^9^, ^10^ ^]^ to material analysis.^[^ ^11^, ^12^ ^]^ Traditional spectrometers have high performance, such as high spectral resolution, sensitivity, and reproducibility, however, their applications are significantly limited due to their cumbersome sizes determined by the intricate optical configurations. This challenge has attracted extensive research focused on developing more compact spectrometers, aiming to achieve reduced size and power consumption, enhanced adaptability, and stability.^[^ ^13^ ^]^ Predominant strategies in this vein include the miniaturization of dispersive optical components, the implementation of narrowband filter‐based spectral detection systems, Fourier transform‐based systems, and systems reliant on computational spectral reconstruction. These advancements in miniaturized spectrometer technology are pivotal in enabling rapid and straightforward spectral detection, especially as spectrometer sizes are reduced to the millimeter scale, thereby laying the groundwork for integrating spectral detection into everyday applications, including integration with smartphones, drones, automobiles, etc.^[^ ^14^, ^15^ ^]^

In the strategies of miniaturization, the computational spectrometer, emerging alongside developments in computational capability and compressive sensing theory,^[^ ^16^, ^17^ ^]^ has attracted notable attention for its minimal spatial footprint and exceptional detection capabilities. This approach involves capturing and encoding spectral data using a limited number of encoding elements, followed by algorithmic reconstruction of the incident light spectrum from these captured signals. Unlike traditional spectrometers that rely on gratings, prisms, or interference for dispersion, computational spectrometers employ diverse encoding methods, such as nanophotonic structures, metamaterials, 2D materials, and micro‐optical elements, significantly reducing device size. Moreover, these spectrometers eschew the need for long optical paths, characteristic of traditional spectrometers for achieving high spectral resolutions, by tightly integrating the encoding structure with the signal detection unit, further compacting their overall sizes. With the development of micro‐nano manufacturing technology and novel materials, more elaborate encoding structures can be produced, facilitating the performance enhancement of computational spectrometers. On the algorithmic side, the rapidly increasing computing power of integrated circuit chips accelerates the reconstruction speed and accuracy.^[^ ^18^ ^]^ Additionally, computational spectrometers are small and power efficient enough to be assembled with computers or smart electronics, taking good use of the robust computing capacities of these platforms for spectrum reconstruction tasks. The operational sequence of a computational spectrometer typically unfolds in three stages: pre‐calibration, spectral information detection, and spectral reconstruction. Pre‐calibration, also referred to as the learning phase, is pivotal for determining the spectral response function of the encoder. This stage is frequently conducted in conjunction with a monochromator or tunable laser. Following this, the spectral information detection phase involves the interaction of incident light with specialized optical structures or directly with the encoded detection elements, resulting in the generation of detection signals. The final stage, spectral reconstruction, leverages both the pre‐calibrated response function and the detection signals. This reconstruction is predominantly executed using compressive sensing algorithms. Nevertheless, with the advent and integration of deep learning algorithms, this workflow has evolved, enabling end‐to‐end neural networks to reconstruct spectra independently, thereby potentially bypassing the pre‐calibration process.

**Figure 1:**
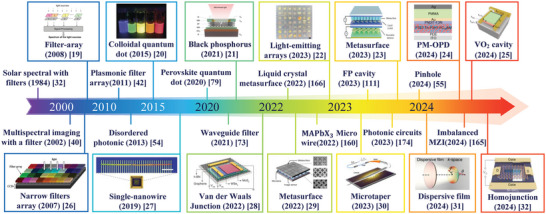
Timeline of advances in miniaturized computational spectrometers. First row of images, left to right: Reproduced with permission.^[^ ^19^ ^]^ Copyright 2008, Optical Society of America; Reproduced with permission.^[^ ^20^ ^]^ Copyright 2015, Nature Research; Reproduced with permission.^[^ ^21^ ^]^ Copyright 2021, Nature Research; Reproduced with permission.^[^ ^22^ ^]^ Copyright 2023, AAAS; Reproduced with permission.^[^ ^23^ ^]^ Copyright 2023, Nature Research; Reproduced with permission.^[^ ^24^ ^]^ Copyright 2024, Nature Research; Reproduced with permission.^[^ ^25^ ^]^ Copyright 2024, Nature Research. Second row of images, left to right: Reproduced with permission.^[^ ^26^ ^]^ Copyright 2007, Optical Society of America; Reproduced with permission.^[^ ^27^ ^]^ Copyright 2019, AAAS; Reproduced with permission.^[^ ^28^ ^]^ Copyright 2022, AAAS; Reproduced with permission.^[^ ^29^ ^]^ Copyright 2022, Wiley; Reproduced with permission.^[^ ^30^ ^]^ Copyright 2023, Springer; Reproduced with permission.^[^ ^31^ ^]^ Copyright 2024, Nature Research; Reproduced with permission.^[^ ^32^ ^]^ Copyright 2024, Nature Research.

As shown in **Figure** **1**, the development of the computational spectrometer can be traced back to the 1980s. Prior to the formal articulation of computational spectrometers, elements of this concept were already applied in the development of multispectral systems for measuring skylights^[^ ^33^, ^34^, ^35^, ^36^ ^]^ and in spectral imaging.^[^ ^37^, ^38^, ^39^, ^40^ ^]^ The emergence of computational spectrometers was inspired by the application of narrowband filter arrays^[^ ^26^ ^]^ and spectral reconstruction methodologies using RGB cameras paired with light‐emitting diode (LED) illumination.^[^ ^41^ ^]^ Furthermore, the idea of spectral reconstruction was also used in a multispectral system, comprising a monochrome charge‐coupled device (CCD) camera and a liquid‐crystal tunable filter.^[^ ^35^ ^]^ The formal introduction of the computational spectrometer as a distinct field occurred in 2008 by the pioneering work of Cheng‐Chun Chang and Heung‐No Lee. They explicitly presented the foundational mathematical model for computational spectrometers and harnessed digital signal processing technology for spectral reconstruction.^[^ ^19^ ^]^

In 2011, Chang et al. discovered the modulating effect of incident light via transmittance,^[^ ^42^, ^43^ ^]^ which could be utilized for spectral encoding. Thus, they proposed a spectrometer based on transmittance modulation, which catalyzed subsequent developments in computational spectrometers^[^ ^44^, ^45^, ^46^, ^47^ ^]^ which attracted increasing attention. In 2015, Bao and Bawendi adopted colloidal quantum dots (QDs) as passive filters and developed a QD spectrometer by coupling the QD filter array with a complementary metal‐oxide‐semiconductor (CMOS) imager, demonstrating the great potential of the computational spectrometer.^[^ ^20^ ^]^

From then on, the computational spectrometer has rapidly evolved, solidifying its status as a groundbreaking paradigm in spectral instrumentation. One of the most remarkable milestones in this journey is the production of ultra‐compact spectrometers, with dimensions in the tens of micrometers range, utilizing a single component gradient nanowire. This advancement is celebrated as a significant stride in the ongoing endeavor to miniaturize spectrometers.^[^ ^27^ ^]^ Recently, computational spectrometers have attracted increasing attention, and some reviews have focused on this promising miniature strategy.^[^ ^48^, ^49^, ^50^, ^51^, ^52^, ^53^ ^]^ While existing reviews have provided valuable insights, they exhibit several limitations that necessitate a more comprehensive perspective. Firstly, most reviews classify spectral encoding methods primarily based on the materials used, lacking a higher‐dimensional viewpoint like the interaction of information with temporal and spatial domains during detection. Secondly, the discussions on spectral decoding are often insufficient, failing to thoroughly analyze this aspect, let alone explore its connection to the characteristics of encoding structures. Finally, the field of miniaturized computational spectrometers is experiencing advancements at an impressive speed, with numerous innovative and inspiring works emerging even within the past year. Therefore, a comprehensive review that integrates both historical context and the latest developments is crucial for summarizing the current state of the field and guiding its future progress.

In this comprehensive review, we summarize the development and recent progress, of miniaturized computational spectrometers, evaluate their performance, and project their future developments. The content is organized according to the two key elements of computational spectrometers: spectral encoding and reconstructive algorithm. The spectral encoding is classified into spatial encoding and temporal encoding, and their corresponding optical structures, materials, and photodetectors are discussed respectively. In the reconstructive algorithm section, the necessary mathematical models are established first, and then the algorithms currently utilized in computational spectrometers are reviewed. Based on the summary of the two elements, we discuss how to cooperate with them and propose performance merits of miniaturized computational spectrometers that are ignored in previous work, outline optimization strategies for computational spectrometry, suggest matters to be taken into account when operating computational spectrometry. The extended applications of miniaturized computational spectroscopy from spectrometry to hyperspectral imaging are also reviewed. At last, foresight into the impending applications and developmental prospects of miniaturized computational spectrometers are presented.

## 2 Spectral Encoding

**Figure 2:**
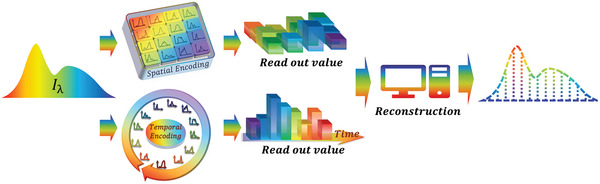
Typical workflow of a computational spectrometer. A typical computational spectrometer comprises a spectral encoding structure through which incident light passes, collected by detectors, and reconstructed. The encoding structure can be classified into spatial and temporal manners.

As depicted in **Figure** **2**, the dominant spectral encoding methodologies in contemporary computational spectrometers predominantly include space‐modulated spectral encoding and time‐modulated spectral encoding. These two distinct approaches reflect a fundamental balance in computational spectrometry between detection duration and spatial requirements. Space‐modulated spectral encoding, specifically, translates the encoding process into the spatial arrangement of the detector array, thereby enabling instantaneous, snapshot‐like spectral sensing. In contrast, time‐modulated spectral encoding necessitates dynamic modulation, utilizing electrical or optical mechanisms, to dynamically alter the spectral response function of the detector. While this method conserves physical space, it prolongs the duration required to complete a single detection cycle. Additionally, light source spectral encoding was also proposed as an interesting strategy by modulating the essential component (light source) when operating spectrometers.

### 2.1 Space‐modulated Spectral Encoding

Spatial modulation in spectral encoding is characterized by the integration of individual detectors with different responses, aimed at achieving a compact spatial encoding of the spectral information. This approach essentially concentrates spectral information into the spatial area occupied by the device. Despite this spatial concentration limits supreme miniaturization, spatial modulation spectrometers still hold a notable advantage in terms of their physical dimensions, especially when compared to traditional, commercially available spectrometers.

Contemporary spatial modulation spectrometers can be generally classified into two main categories based on their strategies for encoding spectral information: speckle‐based spectral encoding and other spectral encoding. Within the realm of spectral encoding, further subdivision is possible based on the design approach of the detector arrays, i.e., indirect filter arrays and direct response arrays.

#### 2.1.1 Speckle‐based Spectral Encoding

**Figure 3:**
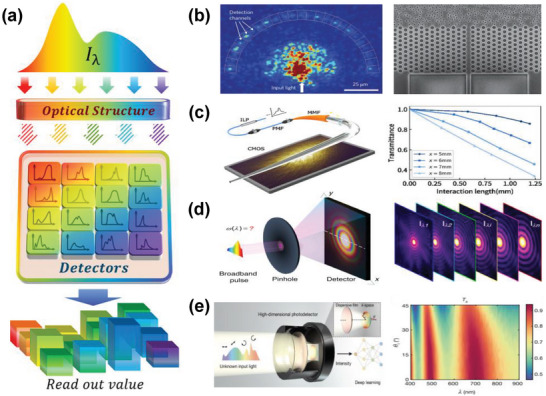
Principle and representative results of speckle‐based encoding. a) The incident light generates different speckle patterns after passing through the optical structure, and the detectors collect signals for spectral reconstruction. b) Speckle generated by scattering from incoherent photonic structures. Reproduced with permission.^[^ ^54^ ^]^ Copyright 2013, Nature Research. c) Speckle generated by a fiber microtaper. Reproduced with permission.^[^ ^30^ ^]^ Copyright 2023, Springer. d) An ultra‐simplified spectrometer based on one‐to‐broadband diffraction speckle. Reproduced with permission.^[^ ^55^ ^]^ Copyright 2024, Nature Research. e) Dispersion‐assisted spectrometer. Reproduced with permission.^[^ ^31^ ^]^ Copyright 2024, Nature Research.

As schematically shown in **Figure** **3**a, a typical computational spectrometer based on speckle encoding typically consists of an optical structure that generates speckle patterns and detectors that detect the light signals. The incident spectrum of light passing through such an optical structure produces one or more speckle patterns based on pre‐designed optical paths. For a single speckle pattern, the detector discretely collects a number of localized or all light intensity signals in the region; for multiple speckle patterns, the light intensity signal is often collected for the whole pattern. Each wavelength corresponds to a distinct spatial speckle pattern with a specific intensity distribution mapping, where the correspondence between the incident wavelength and the generated speckle pattern ensures the possibility of reconstructing the incident light spectrum from the speckle patterns. The principle underlying speckle formation involves incoherent scattering,^[^ ^54^, ^56^, ^57^, ^58^, ^59^, ^60^, ^61^ ^]^ interference,^[^ ^30^, ^62^, ^63^, ^64^ ^]^ diffraction,^[^ ^55^, ^65^, ^66^, ^67^ ^]^ and dispersion.^[^ ^31^, ^68^, ^69^ ^]^ Besides, there has also been work to obtain scattered spots through the rainbow trapping effect.^[^ ^70^ ^]^

The utilization of speckle encoding through incoherent scattering was proposed by Cao, et al. in the year 2013.^[^ ^54^ ^]^ As depicted in Figure 3b, they employed an incoherent photonics chip structure. Through the interaction of incident light with the internal random structures of the chip, multiple reflections and refractions produced 25 distinct speckle patterns at the output end. This design achieved a compact microspectrometer with a small footprint (25 µm radius) and sub‐nanometer resolution (0.75 nm) at a wavelength of 1500 nm. However, out‐of‐plane scattering losses hindered the enhancement of resolution by increasing the size of the incoherent chip. To address this issue, the Cao group introduced a novel approach in 2016, i.e., they utilized spiral waveguides to increase the waveguide length while minimizing additional chip area.^[^ ^63^ ^]^ Due to the introduction of evanescent coupling between adjacent waveguide arms at this stage and the enhancement of non‐resonant behavior, both the bandwidth and resolution of the spectrometer were significantly improved. A spiral spectrometer with a radius of 250 µm was capable of resolving two spectral lines with wavelengths of 1520 nm and a wavelength separation of 0.01 nm. In 2022, Uriel Levy et al. advanced this kind of computational spectrometer by enhancing the spectral resolution to 5 pm.^[^ ^58^ ^]^ They achieved this improvement through the integration of a spiral multimode waveguide interacting directly with guided modes of rubidium vapor and a machine learning algorithm.

Leakage modes in optical fibers are undesirable in their common applications, but it occurred to Ma et al. that the scatter generated by this property could be exploited for spectral encoding and reconstruction, as portrayed in Figure 3c.^[^ ^30^ ^]^ By designing the fiber pulling conditions, they maximize leaky mode generation over a 1 mm taper region and exploit the random interference scattering of the leakage. The final realized spectrometer can achieve a spectral resolution of 1.53 pm in the range of 450–1100 nm with the speckle size shrunk down to an area within 300 µm × 600 µm. This work not only achieves high spectral resolution of the speckle‐based spectrometer but also realizes a broad operating range. With an appropriate image sensor, its working wavelength could be further extended, highlighting a promising potential.

For diffractive speckle encoding, current approaches primarily rely on pinhole arrays with unique pinhole sizes designed to generate distinct diffraction patterns on the surface of a CCD chip. Since each wavelength component in the incident beam corresponds to a unique hole's diffraction angle, the total power received by each pixel on the CCD, after summing the diffraction signals from all wavelength components in the beam, should also be unique. For example, a 10 × 10 hole array fabricated by Ho et al. produces speckles by multiple diffraction and acquires signals through a CCD camera.^[^ ^65^ ^]^ The spectrometer achieves a spectral resolution of 3 nm from 400 to 880 nm and is expected to extend the operating wavelength to the infrared band via the use of an infrared camera. Recently, Gu and Liu et al. developed a spectrometer that doesn't require full‐spectrum calibration, using a simple pinhole as a partial disperser.^[^ ^55^ ^]^ As shown in Figure 3d, this method is based on a unique one‐to‐broadband diffraction decomposition strategy, eliminating the need for complex encoding designs and full spectrum calibration. Through this approach, they achieved sub‐1 nm accuracy in reconstructed spectral peak locations over a bandwidth exceeding 200 nm and remarkable resolution for double peaks separated by 3 nm.

Researchers first employed dispersion in frosted glass to encode spectra. For instance, Ho et al. built a spectrometer based on plain frosted glass and a CCD camera to detect speckle patterns.^[^ ^69^ ^]^ This work realized a spectral resolution of 4.25 nm from 400 to 800 nm. More recently, Jin, Qiu, and Li et al. proposed and demonstrated a miniature photodetector that utilizes a uniform dispersive thin‐film structure for spectral encoding, which is shown in Figure 3e.^[^ ^31^ ^]^ The film is manufactured by standard coating techniques and can be readily used as an alignment‐free retrofit for the existing imaging platforms. By modulating the spatial dispersion at the frequency‐dispersive interface, the detector is capable of characterizing broadband spectra (400–900 nm) and polarization information in a single measurement. Despite the structure is simple, its performance is really excellent, with a spectral resolution of 0.75 nm and full Stokes polarization coverage.

The random media used to generate speckle patterns in the aforementioned contexts are typically artificially created and require high‐precision nanofabrication. Consequently, large‐scale production could potentially face cost challenges. Kim et al. suggested that natural pearls can also provide random speckle patterns for spectral reconstruction.^[^ ^71^ ^]^ The low‐dimensional irregular nanostructures discovered within pearls can be used to create highly uncorrelated spectral resonances through strong light localization, demonstrating spectral compressed sensing as different regions of the pearl exhibit varying speckle patterns and transmission spectra. They selected 16 prominent loci from the speckle patterns originating from the same pearl as their principal data points and the spectrometer attained a spectral resolution of 7.4 nm and featured a system bandwidth spanning 250 nm. This work indicates that low‐dimensional irregular nanostructures can be employed as efficient photonics systems, obviating the use of impeccable and ordered nanostructures. It might be a straightforward and scalable manufacturing strategy for producing compact, cost‐effective, and high‐throughput photonic components.

In summation, present‐day speckle‐based encoding methodologies in spectral sensing exhibit the advantages of high spectral resolution capabilities and small footprints. Nonetheless, many of them suffer from intricate manufacturing processes and restricted operational bandwidths. Besides, a notable characteristic of speckle‐based encoding is its inherent acquisition of complete image information during each operational cycle, which leads to data redundancy, particularly when contrasted with alternative encoding methods. This redundancy may pose a significant constraint, particularly in the pursuit of accelerated real‐time spectral measurements and, more ambitiously, in hyperspectral imaging applications.

#### 2.1.2 Filter Array‐based Spectral Encoding

**Figure 4:**
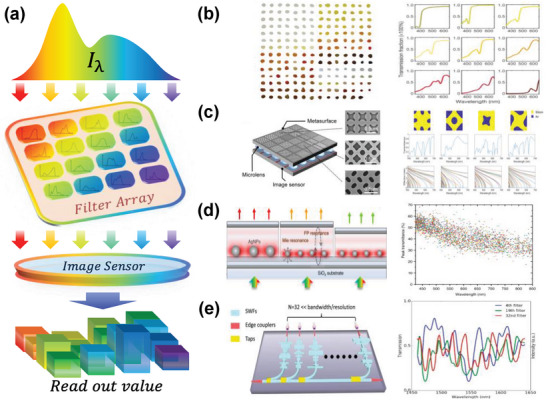
Principle and representative works of filter array‐based spectral encoding. a) The incident light goes through the filter array with each unit having a unique spectral transmission, and the underlying image sensor collects signals for spectral reconstruction. b) Quantum dot‐based optical filters. Reproduced with permission.^[^ ^20^ ^]^ Copyright 2024, Nature Research. c) Metasurfaces‐based optical filters. Reproduced with permission.^[^ ^29^ ^]^ Copyright 2024, Wiley. d) Optical resonant cavities‐based optical filters. Reproduced with permission.^[^ ^72^ ^]^ Copyright 2024, Nature Research. e) Waveguide‐based optical filters. Reproduced with permission.^[^ ^73^ ^]^ Copyright 2021, Nature Research.

As portrayed in **Figure** **4**a, computational spectrometers based on filter array are composed of a set of filters, each with distinct spectral transmittance curves. In this condition, the spectral encoding is solely determined by the optical characteristics of the filters, rather than through optical actions such as scattering in the speckle encoding process. The incident light, after being encoded by the filter array, is typically captured by CCD or CMOS cameras for visible light signals, or by InGaAs image sensors for infrared spectra. Unlike the narrow‐band filtering strategy of miniature spectrometers, the filters in computational spectrometers do not seek narrower passband widths to enhance spectral resolution but rather employ an array of filters with low spectral correlation to each other. Currently, filter arrays designed for computational spectrometers include quantum dots,^[^ ^20^, ^74^, ^75^, ^76^, ^77^, ^78^, ^79^, ^80^, ^81^, ^82^, ^83^, ^84^, ^85^ ^]^ metasurfaces,^[^ ^3^, ^29^, ^86^, ^87^, ^88^, ^89^, ^90^, ^91^, ^92^, ^93^, ^94^, ^95^, ^96^, ^97^, ^98^, ^99^, ^100^, ^101^, ^102^, ^103^, ^104^, ^105^, ^106^ ^]^ resonant cavities,^[^ ^72^, ^107^, ^108^, ^109^, ^110^, ^111^, ^112^, ^113^, ^114^, ^115^, ^116^, ^117^, ^118^, ^119^, ^120^, ^121^, ^122^, ^123^, ^124^ ^]^ and random filters.^[^ ^44^, ^46^, ^47^, ^73^, ^125^, ^126^, ^127^, ^128^, ^129^, ^130^, ^131^, ^132^, ^133^, ^134^, ^135^, ^136^, ^137^, ^138^, ^139^, ^140^, ^141^, ^142^, ^143^ ^]^

Quantum dot filter arrays use tiny QD‐polymer complex filter units, whose absorption onset is tuned by dot size (quantum confinement effect) or composition. Thus, each QD filter unit has a unique transmission spectrum, and the onsets of the spectra in an array cover the spectral range of a QD spectrometer, enabling encoding of the incident light. The QD filter array is featured with the advantages of facile synthetic processes, versatile bandgap engineering capabilities, exceptional optical absorption characteristics, superior photostability relative to conventional dye‐based counterparts, and solution processability. As depicted in Figure 4b, the first QD computational spectrometer was proposed by Bao et al. using 195 distinct CdSe or CdS QD filter units.^[^ ^20^ ^]^ Through deliberate control of the dot size and composition, they achieved continuous fine‐tuning of QD absorption spectra within the spectral range spanning from 390 to 690 nm. However, the spectral resolution of this QD spectrometer approximates only 3.2 nm, which remains insufficient for comparison with the human visual resolution across the majority of the visible light spectrum.^[^ ^144^ ^]^ In 2020, Bian and Zhong et al. adopted MA_3_Bi_2_X_9_ and Cs_2_SnX_6_ (MA = CH_3_NH_3_; X = Cl, Br, I) perovskite QDs to fabricate the filter array, which improves the spectral resolution to 1.6 nm and extends the operational bandwidth (250–1000 nm).^[^ ^79^ ^]^ Notably, this spectral resolution outperforms both the discernment capabilities of human visual perception and the spectral coverage therein. Subsequently, Bian and Zhong et al. achieved a QD infrared spectrometer with an average spectral resolution of 6 nm and a spectral range of 900–1700 nm, using PbS and PbSe QDs.^[^ ^74^ ^]^ Besides, in 2024, Guo and Cao et al. used an inkjet‐printed HgSe QD array to realize an 8–14 µm quantum dot spectrometer, whose spectral resolution reaches 5.4 nm.^[^ ^85^ ^]^ It is imperative to note that current QD spectrometers still lack mature methodologies for large‐scale integrated manufacturing, the limitation of which imposes constraints on their potential for advancement toward quantum dot hyperspectral cameras.

Metasurfaces are optical thin planar arrays of subwavelength scatterers that can be tailored to manipulate various properties of light, including phase, amplitude, polarization, and spectrum. Due to their compact structure, flexible electromagnetic wave control, and compatibility with CMOS manufacturing processes, among other advantages, metasurfaces represent a favorable choice for constructing broadband spatial filter arrays. In 2022, Huang et al. developed a spectral imaging chip based on freeform‐shaped meta‐atoms, thereby breaking the constraints imposed by traditional metasurface designs characterized by regular shapes.^[^ ^29^ ^]^ As portrayed in Figure 4c, they conducted simulations and carefully curated a collection of 400 freeform‐shaped patterns that displayed diverse spectral features due to the complex scattering and coupling behaviors inherent to Bloch modes. Consequently, the chip achieved an impressive spectral resolution of 0.5 nm across the 450–750 nm wavelength range. Furthermore, the manufacturing of metasurface filter arrays can also be based on other materials. In 2019, Yu et al. introduced a random spectrometer based on a photonic crystal slab,^[^ ^89^ ^]^ which achieved a spectral resolution of 1 nm within the range of 550 to 750 nm. In 2022, Crozier et al. fabricated an amorphous silicon‐based metasurface on glass, achieving a spectral response range spanning from 4.75 to 10 µm.^[^ ^92^ ^]^ In 2023, Zheng et al. prepared superconducting nanowire single‐photon detectors (SNSPDs) on metasurfaces with varying structural parameters. They modulated the spectral response of these SNSPDs, thus enabling spatial filtering.^[^ ^87^ ^]^ These works demonstrate the pronounced adaptability in the design and fabrication of metasurface spectrometers, promising numerous avenues for advancing their performance. Moreover, there have been several works demonstrating that metasurfaces can realize spectral reconstruction together with polarization detection, which paves a viable way for high‐dimensional detection.^[^ ^101^, ^102^, ^106^ ^]^ Nevertheless, metasurfaces are confronted with the challenge of signal uniformity, largely influenced by variations in incident angles. Addressing this issue holds paramount importance for the further development of metasurface spectrometers.

Optical resonant cavities leverage the effects of multiple reflections and interference of light waves within the cavity, allowing only specific wavelengths of light to be amplified while suppressing light at other wavelengths. This phenomenon facilitates the realization of spectral encoding through filtering. Early developments based on this principle gave rise to filter arrays such as Fabry‐Perot (FP) filters and plasmonic filters. However, these resonant filters are typically characterized by simple Lorentzian line shapes, representing narrowband filtering characteristics and often lacking in spectral diversity. In order to investigate the influence of this feature on spectral reconstruction performance, Sun et al. conducted spectral restoration simulations using FP resonant cavity filter arrays with varying passband widths.^[^ ^111^ ^]^ The findings demonstrated that narrowband filter arrays excel in reconstructing narrower input signal spectra, whereas the accurate reconstruction of broad‐spectrum inputs is contingent upon the presence of filter arrays characterized by a broader passband peak within the spectral range. Building upon this insight, they engineered filter arrays composed of unit filters that exhibit wider single‐passband peaks alongside narrower multi‐passband peaks, which enabled a spectral resolution of 4 nm within the 450 to 750 nm range.

Due to the inherent periodic characteristics of resonant cavity filters, it is difficult for current optical resonant cavity‐based computational spectrometers to operate over a wide range of wavelengths. Impressively, Wang et al. employed a grayscale photolithography process utilizing a UV laser writing system to integrate 20 FP filter channels on an InGaAs detector chip.^[^ ^110^ ^]^ In the near‐infrared range of 900 to 1700 nm, they achieved a spectral resolution of 5 nm, which not only achieved a wide working range but also broke the Rayleigh criterion in resolution. Lin and Zhang et al. employed a different approach to the broadband operating range, as exemplified in Figure 4d.^[^ ^72^ ^]^ They significantly enhanced spectral diversity by directly printing silver nanoparticles into FP microcavities with varying lengths, leveraging the strong coupling between the nanoparticles' localized surface plasmon resonance and the cavities to modulate the transmission spectra. The work realized a spectral resolution of 0.8 nm within 400–900 nm, which is remarkable among similar strategies.

In addition, micro‐ring resonators can form tunable local samplers through add‐drop filtering.^[^ ^114^, ^123^ ^]^ In 2023, Cheng et al. achieved a computational spectrometer with 256 channels using a cascade of all‐pass micro‐ring resonators, which demonstrated an ultra‐broad bandwidth (>115 nm) and an ultra‐high resolution (<30 pm), paving a new path for on‐chip spectrometers.^[^ ^114^ ^]^

There are two challenges for spectrometers based on resonant cavity arrays. First, it is a complex and difficult task to fabricate the cavity array with each cavity having unique structural parameters. Secondly, the light intensity is substantially reduced due to the inherent optical character of a resonant cavity, which decreases the signal‐to‐noise ratio for the detected electrical signal. This phenomenon becomes particularly pronounced under low‐light conditions, limiting the application scope of this kind of spectrometer.

In addition to the three filter fabrication options mentioned above, a variety of other materials and optical structures have been used for filter encoding, including waveguide arrays,^[^ ^73^, ^129^, ^143^ ^]^ photonic integrated networks,^[^ ^141^, ^142^ ^]^ multilayer thin film structures,^[^ ^126^, ^127^, ^130^ ^]^ gratings,^[^ ^131^, ^145^ ^]^ Linear variable filter,^[^ ^128^ ^]^ multi‐graph filter arrays,^[^ ^132^, ^134^ ^]^ and graded bandgap filter^[^ ^138^, ^139^, ^140^ ^]^ and so on. For instance, as illustrated in Figure 4e, a broadband stratified waveguide filter array with 32 channels was fabricated on a silicon photonics platform,^[^ ^73^ ^]^ whose spectral filters exhibit narrow autocorrelation functions, low inter‐filter cross‐correlation, and achieve a spectral resolution of 0.45 nm over a 180 nm range centered at 1550 nm.

The advantage of spatial filter array encoding in computational spectrometry lies in its ability to capture a wide spectrum of light information simultaneously. This method significantly increases the spectral resolution without the need for narrower passbands. By using a multitude of filters with low correlation, a more detailed and nuanced spectral signature can be obtained. This approach is particularly beneficial for applications requiring high‐throughput analysis and real‐time data processing, as it allows for the rapid acquisition of complex spectral information with a single snapshot.

#### 2.1.3 Detector Array‐based Spectral Encoding

**Figure 5:**
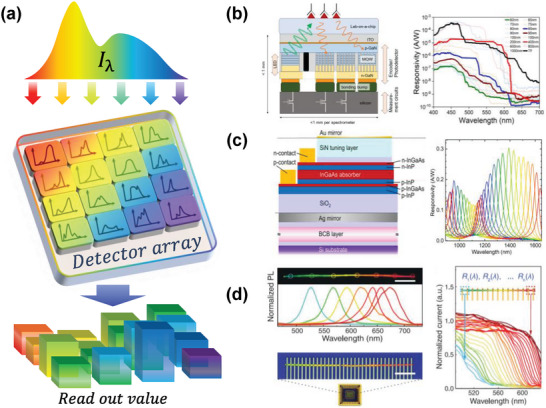
Principle and representative works of spectral encoding based on a detector array. a) The incident light is received by a detector array with each unit having unique spectral responses, and the detectors collect signals for spectral reconstruction. b) A set of gallium nitride nanowires is employed as the absorbers, and the response is modulated by the nanowire diameter. Reproduced with permission.^[^ ^146^ ^]^ Copyright 2022, American Chemical Society. c) Modulating the optical cavity to tune the response of the InGaAs detector. Reproduced with permission.^[^ ^147^ ^]^ Copyright 2023, AIP Publishing. d) Using gradient nanowires to achieve detectors with different spectral responses. Reproduced with permission.^[^ ^27^ ^]^ Copyright 2019, AAAS.

As depicted in **Figure** **5**a, detector array‐based spectral encoding is based on a photodetector (PD) array with each unit having a unique spectral response. This PD array has the same function as the coupling of the above‐mentioned filter array and an image sensor. Thus, the incident light is directly encoded by the PD array without any filter. Based on this direct‐response spectral encoding, the detector array can be primarily categorized into 2D PD arrays^[^ ^146^, ^147^, ^148^, ^149^, ^150^, ^151^, ^152^, ^153^, ^154^, ^155^, ^156^, ^157^, ^158^ ^]^ and 1D PD arrays.^[^ ^27^, ^99^, ^159^, ^160^, ^161^, ^162^, ^163^ ^]^

2D PD arrays use multiple technical routes to fabricate arrays with different spectral responses. For instance, as illustrated in Figure 5b, Gu et al. utilized a set of nanowire gallium nitride nanostructure absorbers as the encoders.^[^ ^146^ ^]^ By employing photolithography and etching techniques to modify the diameter of the nanowires, spectral responses can be programmed. This, combined with the built‐in PN junction, facilitates direct photocurrent measurements, thereby achieving direct spectral response encoding. M. S. Islam et al. developed a spectrometer by employing photon‐trapping surface textures (PTSTs) to engineer the spectral response.^[^ ^158^ ^]^ The PTST‐equipped photodiodes demonstrate high performance with a 27 ps response time, a multiplication gain of 90, and achieve spectral reconstruction from 650–1100 nm. Additionally, there are works that combine filter structures with PDs, as shown in Figure 5c.^[^ ^147^ ^]^ In each detector unit, an InGaAs PD was fabricated inside a resonant cavity. Analogous to resonant cavity structures in filter array‐based encoding, the modulation of cavity length alters the absorption enhancement for different wavelengths across 16 photodetector pixels, covering a spectral range of 890–1650 nm.

Compared to 2D PD arrays, 1D PD arrays offer even greater potential for further reducing the size of spectrometers. For instance, the single nanowire spectrometer developed by Yang et al. in 2019 occupies an area of just 0.5 × 75 µm^2^.^[^ ^27^ ^]^ This spectrometer utilized a CdS_x_Se_1−x_ nanowire with gradient compositions (or bandgaps). Using electron beam lithography, parallel In/Au electrode arrays were fabricated on the nanowires. This configuration results in different spectral response curves at varying nanowire segments, as depicted in Figure 5d. Following a similar manner, Zeng and Xu et al. realized a multispectral sensor operating in the 450–790 nm range with a spectral resolution of 25 nm using a 4 mm gradient bandgap MAPbX_3_ microwire with a diameter of 30 µm.^[^ ^160^ ^]^

Given the spatial limitations imposed by the area of CCD or CMOS image sensors, the development of compact, snapshot‐type computational spectrometers is increasingly favoring responsive encoding strategies over those reliant on filter arrays. However, a crucial consideration for current responsive encoding strategies is the expansion of their operational wavelength range. Presently, many such systems are primarily focused on the visible light spectrum. To fully leverage the potential of responsive encoding, it is essential to broaden this range to other significant spectral regions, such as the near‐infrared and ultraviolet spectra.

### 2.2 Time‐modulated Spectral Encoding

Time‐modulated spectral encoding, a distinct approach within the realm of computational spectrometry, typically employs a limited number of photoresponse tunable detectors, often just one or a few. This method represents a strategic departure from spatial modulation techniques, achieving a further reduction in the physical volume of the spectrometer's detector component. It accomplishes this by effectively compressing the encoding process along the temporal axis, rather than spatially distributing it.

The modification of the detector's spectral response in time‐modulated encoding is generally realized through the use of techniques such as laser pulsing, voltage pulsing, or the application of thermal energy. Each of these methods has its unique mechanisms and implications for the overall design and functionality of the spectrometer. In parallel with the technological advancements in encoding techniques, the miniaturization of these modulating components is also an area of ongoing development, seeking to enhance the compactness and performance of the spectrometers.

Similar to their spatial modulation counterparts, time‐modulated computational spectrometers can be categorized based on the design of their detectors, including those based on tunable filters and those employing detectors with tunable photoresponse.

#### 2.2.1 Tunable filter‐based Spectral Encoding

**Figure 6:**
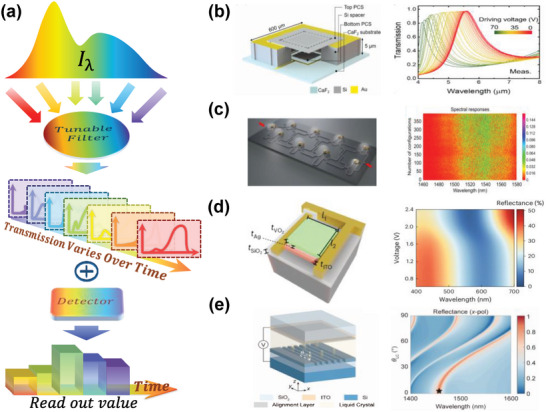
Principle and representative works of spectral encoding based on the tunable filter. a) Incident light passes through a filter structure with adjustable spectral transmission, and the detector collects signals for spectral reconstruction. b) Electrostatically driven FP filter with adjustable resonance wavelength. Reproduced with permission.^[^ ^164^ ^]^ Copyright 2021, Elsevier. c) Power‐tuned photonic integrated circuits. Reproduced with permission. Copyright 2024, Opto‐Electronic Advances. d) Voltage‐tuned vanadium dioxide(VO_2_) cavities.^[^ ^25^ ^]^ Copyright 2024, Nature Research. e) Voltage‐regulated liquid crystal metasurface. Reproduced with permission.^[^ ^165^ ^]^ Copyright 2022, Springer.

As shown in **Figure** **6**a, a spectral encoding system based on a tunable filter generally comprises a single filter with adjustable transmittance and a photodetector. During measurement, the transmittance curve of the tunable filter used for encoding undergoes significant changes by applying laser pulsing, voltage pulsing, or the application of thermal energy. Consequently, the measured light intensity signal varies with time, thus accomplishing spectral encoding in the time dimension. Presently, tunable filters for computational spectrometers primarily utilize resonant cavity structures, including FP cavities,^[^ ^164^, ^166^, ^167^, ^168^ ^]^ phase‐change cavities,^[^ ^25^, ^169^ ^]^ plasmonic resonators,^[^ ^170^ ^]^ waveguide resonators,^[^ ^171^, ^172^ ^]^ and integrated photonic network.^[^ ^173^, ^174^, ^175^ ^]^ Besides, liquid crystal and tunable metasurface can also be used as tunable filters.^[^ ^165^, ^176^, ^177^, ^178^, ^179^ ^]^

In 2021, Lee et al. developed a computational spectrometer operating in the mid‐wave infrared range. It utilized a textile‐based triboelectric nanogenerator to provide a high open‐circuit voltage for tuning the resonance wavelength of an electrostatically driven FP cavity.^[^ ^164^ ^]^ As shown in Figure 6b, the FP resonance peak shifted from 5.65 to 5.33 µm, completing the encoding process.

Dai et al. achieved a super‐high‐resolution computational spectrometer by cascading a thermally tunable ultra‐high‐Q resonator with an N‐channel broadband resonator array.^[^ ^171^ ^]^ The unknown input spectrum is initially pre‐filtered by the ultra‐high‐Q resonator, with its resonance wavelength corresponding to the applied heating power. Subsequently, it is divided into N sub‐spectra by the N‐channel broadband resonator. As the heating power is scanned, the transmittance of each subsection of the sub‐spectrum can be sequentially retrieved from the output port of the respective broadband resonator. During the scan, the power ranges from 0 to 50.4 mW, with fine steps of 0.029 mW, and a scan time step of ≈1 ms. The total duration is ≈2 s, which still falls short of the real‐time imaging speeds required for human visual perception.

Using standard integrated circuit processes on silicon substrates, the integrated photonic network‐on‐chip can be efficiently manufactured.^[^ ^173^ ^]^ Based on this platform, Cheng et al. not only created a spectrometer based on spatial encoding^[^ ^114^ ^]^ but also further developed a time‐encoded spectrometer.^[^ ^173^ ^]^ This spectrometer utilizes a 6‐stage cascaded Mach‐Zehnder Interferometer (MZI) design. These MZI components are manufactured on silicon substrates using precise photolithography and etching techniques and utilize Thermo‐Optic Phase Shifters to adjust the bias voltage for phase control, thus achieving different low‐correlation spectral responses. With a bandwidth exceeding 200 nanometers, this spectrometer uses 729 sampling channels to achieve a single measurement duration of 0.8 seconds and a resolution of <10 picometers. Similarly, Yang and Pan et al. presented a spectrometer with a 2D array of imbalanced Mach‐Zehnder Interferometers interconnected in a mesh configuration.^[^ ^174^ ^]^ As shown in Figure 6c, the spectral transmittance of the system can be efficiently modified by injecting a random amount of power into the 11 phase shifters used. By choosing the number of phase shifters, the spectrometer is capable of switching between a high‐power mode with 10 pm resolution and a low‐power mode with 0.3 nm resolution in the 1460–1580 nm range.

Electrically driven phase change materials have enabled significant breakthroughs in tunable nanophotonics.^[^ ^180^, ^181^ ^]^ Recently, He et al. developed a phase‐change resonant cavity spectrometer with high‐speed tuning capabilities using VO_2_, as displayed in Figure 6d.^[^ ^25^ ^]^ This system can withstand millions of cycles without significant performance degradation at a voltage modulation rate of 70 kHz, far surpassing other existing methods. Additionally, spectral imaging was achieved in the 400–700 nm range using four filters per spectral pixel, with a root mean square error (RMSE) below 0.05 for narrowband, 0.06 for broadband imaging and the maximum peak difference (Δλ) kept under 2.5 nm.

In addition, a tunable graphene plasmonic filter was also applied to encode the incident spectrum. By altering the gate voltage and thus modifying the Fermi energy of the midgap graphene plasmon, the resonance wavelength can be changed, enabling tunable filters.^[^ ^170^ ^]^ This work achieved a spectral resolution of <100 nm within the wavelength range of 8 to 14 µm.

Tunable metasurfaces have emerged as important optical structures,^[^ ^182^ ^]^ which can be adopted for spectral encoding. Notably, computational spectrometers based on liquid crystal modulation of metasurface were realized in 2022 by Yang et al., as depicted in Figure 6e.^[^ ^165^ ^]^ In this work, they embedded the metasurface into the liquid crystal layer and applied alternating current through Indium tin oxide electrodes. When the incident light is polarized along the x‐axis, a significant change in the liquid crystal's refractive index occurs, enabling temporal encoding. This spectrometer exhibits excellent reconstruction performance for narrowband signals with a minimum Full Width at Half Maximum (FWHM) of 2 nm in the range of 1400–1500 nm and requires only 60 ms for a single measurement. In addition to its combination with metasurfaces, the liquid crystal can also be used in the manufacture of delay lines to achieve voltage‐tunable filtering.^[^ ^35^, ^45^, ^183^, ^184^ ^]^ Their work was improved in 2016,^[^ ^176^, ^177^ ^]^ and furthermore, they realized a 4D camera that could efficiently capture 3D spatial images together with their spectral information in 2019.^[^ ^178^ ^]^

The spectral encoding method utilizing a tunable filter provides a dynamic approach to spectral reconstruction. Compared to spatial filter array encoding, this method allows for a more compact system design. However, it may also introduce challenges, such as the need for precise control of external stimulus and potential limitations in the tuning response speed, which could impact the overall data collection time for spectral detection.

#### 2.2.2 Tunable Photodetector‐based Spectral Encoding

**Figure 7:**
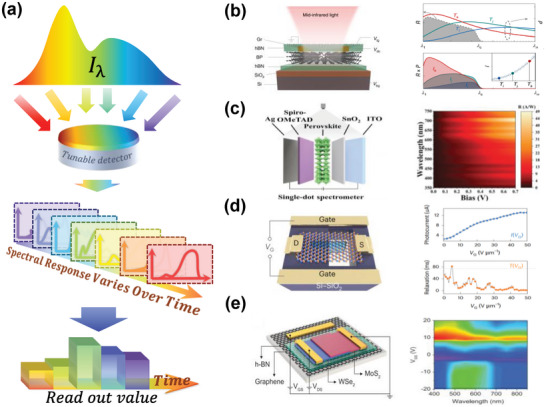
Principle and representative works of spectral encoding based on tunable photodetector. a) Incident light is received by a detector with an adjustable spectral response and the signals are collected at different times for spectral reconstruction. b) A spectrometer based on a voltage‐tunable black phosphorus detector. Reproduced with permission.^[^ ^21^ ^]^ Copyright 2021, Nature Research. c) A spectrometer based on a voltage‐tunable perovskite detector. Reproduced with permission.^[^ ^185^ ^]^ Copyright 2022, Wiley. d) A spectrometer based on semi‐floating molybdenum disulfide homojunction. Reproduced with permission.^[^ ^32^ ^]^ Copyright 2024, Nature Research. e) A spectrometer based on a van der Waals heterojunction. Reproduced with permission.^[^ ^28^ ^]^ Copyright 2022, AAAS.

Similar to the tunable filter, PDs, whose photoelectric response can be tuned by heat or electricity, are also employed for spectral encoding, as shown in **Figure** **7**a. This strategy is similar to the above‐mentioned PD array‐based encoding but changes the spatial encoding to encoding in the time dimension with a single PD. This contributes to further advancements in system compactness, thereby garnering increasing attention.^[^ ^21^, ^24^, ^28^, ^32^, ^185^, ^186^, ^187^, ^188^, ^189^, ^190^, ^191^, ^192^, ^193^, ^194^ ^]^

As shown in Figure 7b, Xia et al. developed the first miniature computational spectrometer based on a single photodetector.^[^ ^21^ ^]^ This work achieved tunable spectral response encoding through a 13 nm thick black phosphorus (BP) film, with this thickness ensuring that the film can absorb light effectively while avoiding hindrance to bandgap adjustment. The BP spectrometer adjusted its bandgap by applying a voltage to the source‐drain electrodes made of chromium/gold, achieving a spectral resolution of 0.42 µm with 41 sampling points in the range of 1 to 9.5 µm^2^. The active area footprint of this spectrometer is only 9  ×  16 µm and its spectral resolution can be further improved by reducing scanning range and increasing sampling points.

Organic‐inorganic hybrid perovskites are a widely investigated semiconductor material known for high charge carrier mobility, suitable bandgaps, and adjustable composition and band structure, making them ideal materials for spectral response modulation. Compared to the spatial‐spectral encoding using perovskite quantum dots,^[^ ^79^ ^]^ Li and Sun et al., based on a 5% LiCl‐doped perovskite film, have developed a single‐detector computational spectrometer that can achieve spectral reconstruction with a small footprint, as illustrated in Figure 7c.^[^ ^185^ ^]^ By applying an external electric field to change the bias, they attained photogain manipulation controlled by ion redistribution in the perovskite film, thus, modulating the spectral response of the device, and achieving spectral encoding. This time‐modulated spectrometer demonstrated good response speed, with a stable response time of <25 µs for each change. Ultimately, it obtained a resolution of 5.3 nm with 97 sampling points in the range of 350–550 nm. Similarly, Hao et al. fabricated a perovskite single‐phototransistor spectrometer, which increased the spectral resolution to 1 nm using 400 sampling points.^[^ ^188^ ^]^

Solution‐processed organic materials enable the fabrication of low‐cost, large‐area optoelectronic devices and have been explored for image sensor applications.^[^ ^195^, ^196^ ^]^ However, for the use of computational spectrometers, an efficient mechanism to dynamically tune their spectral response is essential. As displayed in Figure 7d, Zhao et al. have proposed a novel solution to this task.^[^ ^24^ ^]^ They combined the contact with an organic ternary active layer and a back‐to‐back Schottky diode design by integrating an optical spacer with an additional back‐reflector, which allowed the heterojunction to have an optical range difference of sufficient length to selectively drive carriers to the contact in a wavelength‐dependent manner with the interfacial energy bands bent to different extents at different bias voltages. The final spectrometer obtains a spectral resolution of <5 nm within 400–760 nm and occupies only 0.0004 cm^2^.

Van der Waals heterostructures formed between different 2D transition metal dichalcogenides exhibit a type II band alignment, providing a universal platform to explore interlayer excitons. Due to the fact that the conduction band minimum and valence band maximum of the heterostructure are distributed in different layers, this typical type II heterostructure opens up a tunable degree of freedom in the infrared region. It allows for interlayer optical transitions beyond the intrinsic optical bandgaps of the constituent materials, making it suitable for responsive and tunable spectral encoding. As shown in Figure 7e, Sun and Yoon et al. used MoS_2_/WSe_2_ heterostructures to create a micro‐spectrometer with photocurrent response varied under different gate voltages,^[^ ^28^ ^]^ where they achieved a spectral resolution of 3 nm within the range of 405–845 nm. In 2024, Sun et al. developed a high‐performance broadband spectrometer based on a van der Waals heterostructure diode composed of BP/MoS_2_, which operates from ≈500 to 1600 nm and has a resolution of 2 nm.^[^ ^189^ ^]^ Similarly, Duan and Zhang et al. developed and demonstrated a micro‐spectrometer based on the ReS_2_/WSe_2_ heterostructure.^[^ ^187^ ^]^ They achieved spectral reconstruction with a resolution of 20 nm within the range of 1150–1470 nm; Wu et al. utilized a single SnS_2_/ReSe_2_ van der Waals heterostructure and realized a spectral resolution of 5 nm with a bandwidth from 400 to 800 nm;^[^ ^190^ ^]^ Hu et al. utilizes a 2D/3D van der Waals heterostructure (p‐type germanium/n‐type molybdenum disulfide/p‐type black phosphorus) photodetector to achieve a miniaturized computational spectrometer covering the range of 500–2000 nm.^[^ ^192^ ^]^ These studies demonstrate the feasibility of micro‐spectrometers based on van der Waals heterostructures for the development of computational spectrometers relying on a single detector. They provide a promising approach for miniaturizing spectrometers and exploring compact solutions.

Fabricating heterojunctions from 2D materials for miniaturized computational spectrometers is common because of the ease of tuning their optoelectronic response by adjusting the materials' bandgaps. Homojunctions, however, are more challenging as it is more difficult to acquire varied spectral responses. Recently, Cui, Zhao, and Xiong et al. demonstrated a semi‐floating molybdenum disulfide homojunction, leveraging its electrostriction effect to modulate the bandgap and tune the spectral response.^[^ ^32^ ^]^ They further enhanced spectral reconstruction by introducing a nonlinear encoding route, i.e., response time. They achieved this by chopping the incident light at a fixed frequency, scanning the in‐plane gate voltage, and extracting the photocurrent signal through a transconductance amplifier. This approach of employing two dimensions of coded information yields a spectral resolution of 1.2 nm in the 450–800 nm range, with a device footprint of only 20 × 25 µm^2^.

In addition to the above strategies, Yu and Chen, et al. developed a miniaturized spectrometer using a p‐graded‐n junction with voltage modulation.^[^ ^194^ ^]^ Unlike the three‐terminal device structure commonly used in heterostructures of 2D‐based materials, their design employs a two‐terminal configuration comprising a gradient bandgap Al_x_Ga_1−x_As n‐layer with different Al composition, meaning the physical lateral size of the device does not affect its functionality, thus offering better integration potential. This work achieves wavelength peak accuracy of up to 0.3 nm and a spectral resolution of 10 nm within 480–820 nm, with detector diameter varying from 20µm to 2 mm.

Spectral encoding strategies based on response‐tunable PDs are expected to result in the smallest size of the spectrometer compared to all other spectral encoding strategies because only a single detector is used. In addition, there is no issue related to incident angle existing in filter‐based strategies. However, this strategy has a high requirement for the repeatability of the tunable PD.

### 2.3 Light Source Spectral Encoding

**Figure 8:**
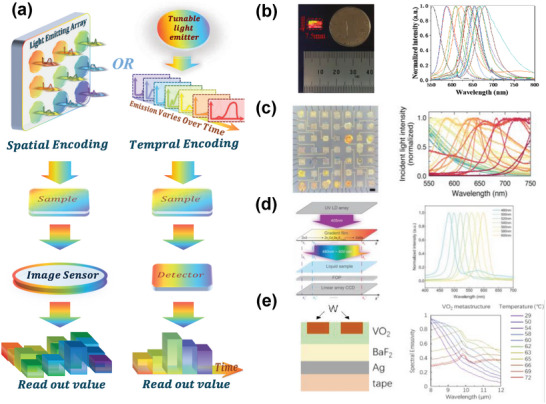
Principle and representative works of light source spectral encoding. a) Incident light is generated by a light‐emitting array or adjustable light emitter, which passes through the sample and is subsequently collected by an image sensor or a detector for spectral reconstruction. b) QD array composed of QDs with different sizes to emit light with different peaks. Reproduced with permission.^[^ ^197^ ^]^ Copyright 2023, Optica Publishing Group. c) Multiple light emitters and pulse modulation are employed to obtain diverse emission spectra. Reproduced with permission.^[^ ^22^ ^]^ Copyright 2023, AAAS. d) A bandgap‐graded semiconductor nanofilm. Reproduced with permission.^[^ ^198^ ^]^ Copyright 2024, IOP Publishing. e) The use of thermally tunable VO_2_ material via phase change to generate varied emission spectra. Reproduced with permission.^[^ ^199^ ^]^ Copyright 2024, Optica Publishing Group.

Light source is essential during the spectral measurement, therefore, in addition to the previous spectral encoding based on modulation of filters or detectors, the modulation of light source emerges as a new spectral encoding strategy, recently. This design allows the light source to emit a variety of known spectra, effectively completing the encoding process, as depicted in **Figure** **8**a. Each light source unit emitting a spectrum with different peaks is recorded by a fixed detector in the pre‐calibration process, and the sample is placed between the light source and the detectors to measure the spectrum in a transmission manner. Similar to the previous spectral encoding methods, the light source encoding can employ both spatially coded array emission and temporally coded tunable emission strategies, as shown in Figure 8a.

The encoding work in light source‐based systems has traditionally utilized LED illumination methods.^[^ ^200^, ^201^ ^]^ A significant challenge associated with this approach, however, is the limited diversity of the spectra that these LEDs can generate, often resulting in suboptimal spectral reconstruction outcomes. Recently, a variety of other materials were employed to improve the light source encoding. For instance, Wang et al. employed a QD array composed of QDs of different sizes as the light source. Due to the confinement effect, these QDs emitted narrowband light within specific wavelength ranges.^[^ ^197^ ^]^ Here, as shown in Figure 8b, the QD array inherently fulfills the dual roles of serving as both the light source and the wavelength‐splitting structure, thereby obviating the necessity for supplementary wavelength‐splitting components. This technological advancement holds the promise of substantially diminishing the volume of spectrometers equipped with integrated light sources. The spectrometer, operating within the 580–720 nm range, attained a spectral resolution of 9.7 nm.

Besides QDs, other emissive materials, such as organic small molecules, and conjugated polymers are used for spectral encoding.^[^ ^22^ ^]^ In this way, a dense library of emission spectra is prepared by depositing emissive materials atop a capacitor and exciting luminescence with voltage pulses across the visible light range. As depicted in Figure 8c, the generated large‐scale multicolor electroluminescent array not only exhibited diverse spectral for each unit but also enabled each light‐emitting unit to tune the intensity by applying voltage pulses of varying frequencies. The authors show the potential of producing light spectra covering the range of 400–1400 nm.

Bandgap‐graded film can be employed for filter array‐based spectral encoding when placed on the detection side,^[^ ^138^, ^139^, ^140^ ^]^ and it can also be used to modulate the light source when placed on the light‐emitting side. Yang et al. followed this route and realized a spectral detect system in a chip, which integrates a linearly tunable light source, sample chamber, and detector.^[^ ^198^ ^]^ By stimulating different regions of a ZnCdSeS bandgap‐graded semiconductor nanofilm with UV light, a linearly tunable light source is generated, covering a wavelength range from 480 nm to 600 nm.

In addition to modulating the light source spatially, temporal encoding is also applied. As illustrated in Figure 8e, Wu et al. fabricated a tunable mid‐infrared (MIR) light source based on the phase transition of VO_2_ by controlling the temperature.^[^ ^199^ ^]^ Then the authors demonstrated a single‐pixel reconstructive MIR spectrometer and measured the transmission spectrum of a magnesium fluoride sample.

The light source modulation provides a new idea for spectral encoding, however, it faces some challenges. First, the spectrometers based on light source spectral encoding usually operate in a transmission manner, requiring samples without scattering and limiting their applications. Second, it is difficult to achieve efficient emissions for all the materials or micro‐devices. Third, the stability of the emissive materials and devices is another challenge.

Based on the above analysis, the characters of the mentioned spectral encoding structures and the performance of their spectrometers are summarized in **Table** **1** for comparison.

**Table 1:** Typical works of computational spectrometers and their performance.

Encoding strategy		Structure	Spectral range/bandwidths	Resolution	Footprint	Modulating speed
Space‐modulated spectral encoding	Speckle encoding	Disordered photonics chip.^[^ ^54^ ^]^	1500–1525 nm	0.75 nm	25 µm radius	
		Fiber microtaper^[^ ^30^ ^]^	450–1100 nm	1.53 pm	N/A	
		A pinhole^[^ ^55^ ^]^	200 nm	3 nm	N/A	
		Dispersive thin‐film^[^ ^31^ ^]^	400–900 nm	0.75 nm	N/A	
	Filter array‐based spectral encoding	Quantum dots^[^ ^20^ ^]^	390–690 nm	3.2 nm	N/A	
		Metasurfaces^[^ ^29^ ^]^	450–750 nm	0.5 nm	N/A	
		Optical resonant cavities^[^ ^111^ ^]^	450 to 750 nm	4 nm	N/A	
	Detector array‐based spectral encoding	Gallium nitride nanowires^[^ ^146^ ^]^	400–645 nm	N/A	0.16 mm^2^	
		Optical resonant cavities^[^ ^147^ ^]^	890–1650 nm	N/A	1.4 × 1.4 mm^2^	
		Gradient nanowires^[^ ^27^ ^]^	500–630 nm	15 nm	0.5 × 75 µm^2^	
Time‐modulated spectral encoding	Tunable filter‐based spectral encoding	Power‐tuned photonic integrated circuits^[^ ^174^ ^]^	1460–1580 nm	10 pm	N/A	N/A
		Thermal tunable resonators^[^ ^171^ ^]^	10 nm	5 pm	0.35 mm^2^	2 s
		Voltage‐tuned VO_2_ cavities^[^ ^25^ ^]^	400–700 nm	N/A	N/A	70 kHz
		Voltage‐regulated liquid crystal metasurface^[^ ^165^ ^]^	1400–1500 nm	N/A	N/A	60 ms
	Tunable photodetector‐based spectral encoding	Voltage‐tunable black phosphorus^[^ ^21^ ^]^	1–9.5 µm	0.42 µm	9 × 16 µm^2^	N/A
		Voltage‐tunable organic photodetector^[^ ^24^ ^]^	400–760 nm	sub‐5 nm	0.0004 cm^2^	197 µs
		Molybdenum disulfide homojunction^[^ ^32^ ^]^	450–800 nm	1.2 nm	20 × 25 µm^2^	N/A
		Van der Waals heterojunction^[^ ^28^ ^]^	405–845nm	3 nm	N/A	N/A
Light source spectral encoding		Quantum dots^[^ ^197^ ^]^	580–720 nm	9.7 nm	4 × 7.5 mm^2^	
		Multicolor electroluminescent array^[^ ^22^ ^]^	550–750 nm	N/A	N/A	
		Phase transition of VO_2_^[^ ^199^ ^]^	8–12 µm	N/A	N/A	N/A

## 3 Reconstruction Algorithms

In the previous sections, we focus on how computational spectrometers compress and encode spectral information through various methods. Efficient coding methods are essential for the implementation of computational spectrometers. The performance ceiling is determined by both the efficiency of individual coding units and the total number of units employed. Higher coding efficiency allows for fewer units, minimizing time and spatial resources. On the other hand, the capability of the reconstruction algorithm to accurately retrieve spectral information from the encoded signal dictates the spectrometer's ability to reach its performance limits. Typically, the problems to be addressed by the reconstruction algorithm can be formulated as:

(1) $$\int_{\lambda_{min}}^{\lambda_{max}}F\left(\lambda \right)R_{i}\left(\lambda \right)d\lambda =b_{i}\;\left(i=1,2,3,\text{…}n\right)$$

where the integral process signifies the encoding procedure of the spectrum, *R_i_* (λ) represents the encoding function of each encoding element within the operational wavelength range $\lambda_{min}∼\;\lambda_{max\;}$, *F*(λ) denotes the spectrum of the incident light to be reconstructed, *b_i_* corresponds to the signal received by the detector, and *n* indicates the number of obtained codings.

Typically, *b_i_* is directly read out by the detector, while *R_i_* (λ) necessitates scanning with monochromatic light within the operational range. During this scanning process, monochromatic light often exhibits a Gaussian distribution rather than an ideal single beam, thus allowing *F*(λ) to be approximated as

(2) $$F\left(\lambda \right)\approx \sum_{j=1}^{m}\phi_{j}\left(\lambda \right)x_{j}$$

here $\phi_{j}\left(\lambda \right)=\frac{1}{\sigma \sqrt{2\pi }}\exp\left[-\frac{1}{2}{\left(\frac{\lambda -\hat{\lambda_{j}}}{\sigma }\right)}^{2}\right]$, where a Gaussian function ϕ_*j*_(λ) is employed to describe the distribution of monochromatic light.^[^ ^202^, ^203^ ^]^ The parameter $\sigma ={\left(2\sqrt{ln2}\right)}^{-1}\delta_{d}$, with δ_*d*_ representing FWHM of the actual monochromatic light, $\hat{\lambda_{j}}$ indicating the central wavelength of the monochromatic light for the *j_th_* scan, and *j* signifies the number of monochromatic lights in the scanning process. Therefore, the problem to be addressed can be reformulated as:

(3) $$\sum_{j=1}^{m}\left(\int_{\lambda_{min}}^{\lambda_{max}}R_{i}\left(\lambda \right)\phi_{j}\left(\lambda \right)d\lambda \right)\;x_{j}=b_{i}\left(i=1,2,3,\text{…}n\right)$$

which can be succinctly represented as a matrix equation:*Ax*  =  *b*, where *A* is a known *n* × *m* matrix, and $A_{ij}=\int_{\lambda_{min}}^{\lambda_{max}}R_{i}\left(\lambda \right)\phi_{j}\left(\lambda \right)d\lambda ,\;b={\left[b_{1},b_{2},\;\text{…},b_{n}\right]}^{T}$. The decomposition of incident light in this model is not applicable to all algorithms, especially end‐to‐end neural networks, which do not require access to the spectral encoding structure itself for reconstruction, but rather the encoded signals and its corresponding input spectrum. The adoption of a compression encoding strategy in computational spectrometers invariably leads to a result that *n* ≪ *m*, leading to the matrix *A* being underdetermined. This underdetermined nature of the equation poses a unique challenge in the field of spectral reconstruction, necessitating specialized algorithms capable of deriving reliable and effective solutions.

Presently, the algorithms used for spectral reconstruction in spectrometers can be generally classified into two categories: traditional reconstruction algorithms and deep learning algorithms. Traditional reconstruction algorithms have been the mainstay for some time and include methods like sparse coding, basis pursuit, and compressive sensing techniques. These algorithms have been empirically validated for their effectiveness in dealing with underdetermined equations, especially in the context of spectral data reconstruction. On the other hand, deep learning algorithms, which leverage extensive datasets for training, have emerged as a powerful alternative. These algorithms harness the capabilities of neural networks to model complex relationships and patterns within data, offering significant advantages in terms of reconstruction accuracy and efficiency. Moreover, deep learning techniques are not only used for the reconstruction process but have also been increasingly applied in reverse engineering the encoding structure of the spectrometer. This application allows for a synergistic optimization of both the software (algorithmic) and hardware components of the computational spectrometers, leading to enhanced overall performance.

### 3.1 Traditional Reconstruction are Construction Algorithms

The traditional algorithms have undergone thorough investigation and found applications in diverse domains, including computational spectrometers image denoising, etc. Basically, computational reconstruction and applications such as image denoising share a common objective, i.e., reconstructing the original signal from its sparse representation. Consequently, since the emergence of computational spectrometry, researchers recognized the potential of seamlessly transitioning the classical algorithms from image denoising to this emerging field. Actually, the long‐established algorithms have demonstrated excellent performance in the task of computational reconstruction for spectrometers, substantially accelerating their development.

**Figure 9:**
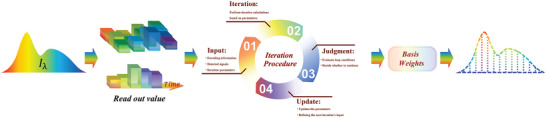
Procedure of reconstruction strategy based on traditional algorithms.

As illustrated in **Figure** **9**, traditional reconstruction algorithms proceed through a four‐step sequence: input preparation, iterative computation, convergence assessment, and parameter update. The process begins with the preparation of input parameters, which typically include detected signals, processed encoding information, and algorithm‐specific iteration parameters. Subsequently, the algorithm engages in iterative computation, executing the necessary calculations as prescribed by the model. Following this, the results are subjected to a convergence assessment, where they are evaluated against predefined criteria, such as a threshold or convergence condition, to determine if further iterations are warranted. Should additional iterations be necessary, the parameters are updated and integrated with the initial inputs, preparing the system for the next cycle. Upon meeting the convergence criteria, the final task involves reconstructing the spectral information using basis functions, such as ϕ in Equation 2, with the corresponding basis weights obtained from the iterative process.

As summarized in **Table** **2**, the traditional algorithms applied in computational spectrometry are analyzed. These algorithms are classified into the least squares algorithm and its improved algorithms, regularization techniques, as well as some other optimized computation strategies.

**Table 2:** Traditional reconstruction algorithms in current computational spectrometers.

Traditional Reconstruction Algorithms		Mathematical Feature
Least Squares and Improved Algorithms	Generalized Least Squares Algorithm^[^ ^20^, ^94^, ^118^, ^150^, ^199^ ^]^	$\min_{x}\frac{1}{2}∥Ax-b{∥}^{2}$ *x* ^*k* + 1^ = *x^k^* − *tA^T^* (*Ax^k^* − *b*)
	Levenberg‐Marquardt Algorithm^[^ ^185^ ^]^	*x* ^*k* + 1^ = *x^k^* + Δ $\Delta =-{\left(J_{f}^{T}J_{f}+\lambda I\right)}^{-1}J_{f}^{T}f$
	Recursive Least Squares Algorithm^[^ ^86^, ^92^, ^99^, ^125^, ^187^ ^]^	$y^{k+1}=\frac{1}{\lambda }y^{k}-\frac{1}{\lambda }\eta^{k}x^{kT}y^{k}$ *A* ^*k* + 1^ = *A^k^* − η^*k* + 1^(*x^kT^A^k^* − *b* ^*k* + 1^)
Regularization Techniques	Lasso Regularization^[^ ^21^, ^63^, ^71^, ^93^, ^99^, ^127^, ^145^, ^169^, ^190^ ^]^	$\min_{x}\frac{1}{2}∥Ax-b{∥}^{2}+\lambda ∥x∥$ $x^{k+1}=x^{k}-t\left[A^{T}\left(Ax^{k}-b\right)+\lambda \frac{x}{∥x∥}\right]$
	Tikhonov Regularization^[^ ^21^, ^24^, ^27^, ^28^, ^55^, ^73^, ^102^, ^108^, ^119^, ^124^, ^138^, ^139^, ^154^, ^155^, ^160^, ^164^, ^166^, ^167^, ^189^, ^190^ ^]^	$\min_{x}\frac{1}{2}∥Ax-b{∥}^{2}+\frac{\lambda }{2}∥x{∥}^{2}$ *x* ^*k* + 1^ = *x^k^* − *t*[*A^T^* (*Ax^k^* − *b*) + λ*x^k^*]
	Total Variation Regularization^[^ ^45^, ^74^, ^79^, ^83^, ^104^, ^122^, ^146^, ^152^ ^]^	$\min_{x}\frac{1}{2}∥Ax-b{∥}^{2}+\lambda ∥\nabla x∥$ *x* ^*k* + 1^ = *x^k^* − *t*[*A^T^* (*Ax^k^* − *b*) + λ∇(∇*x*)]
Other Traditional Algorithms	Accelerated Proximal Gradient Algorithm^[^ ^108^, ^110^ ^]^	*y* ^*k* + 1^ = *x^k^* + ω^*k*^(*x^k^* − *x* ^*k* − 1^) $x^{k+1}=prox_{\lambda^{k}g}\left(y^{k+1}-t^{k}\nabla f\left(y^{k+1}\right)\right)$
	Heuristic Algorithms^[^ ^54^, ^96^, ^133^, ^142^ ^]^	Simple Heuristic Algorithms Meta‐Heuristic Algorithms
	Prefiltering Algorithms^[^ ^57^, ^132^, ^134^, ^179^ ^]^	$\min_{x}\frac{1}{2}∥Ax-C{∥}^{2}$ *C* = b*g_filter_

#### 3.1.1 Least Squares and Improved Algorithms

For the least squares algorithm and its improved algorithms, the most common one is the generalized least squares (GLS) algorithm,^[^ ^20^, ^94^, ^118^, ^150^, ^199^ ^]^ which employs gradient descent as the iterative criterion. This algorithm transforms the original underdetermined equations using the *l_2_* norm, turning it into a convex optimization problem for resolution, as indicated in Table 2. However, the drawback of GLS lies in its high sensitivity to noise.^[^ ^79^ ^]^ When the noise level reaches a certain threshold during the hardware encoding process, the reconstruction performance of this algorithm experiences a notable deterioration.

Differing from GLS, the Levenberg–Marquardt algorithm (LMA) primarily enhances the iterative approach.^[^ ^185^ ^]^ Derived from the Gauss‐newton method, LMA introduces a coefficient *λ* and adjusts the descent rate according to the trust‐region principles, thereby reducing the number of iterations. However, there is no conspicuous improvement in its noise tolerance performance.

Recursive least squares (RLS) are designed specifically to address situations where the vector *b* in the matrix equation cannot be obtained at once, resulting in the problem that the iteration can't be handled in one single batch.^[^ ^86^, ^92^, ^99^, ^125^, ^187^ ^]^ In the RLS algorithm, the model can adaptively update itself upon acquiring new data, reducing computational complexity. This pipeline‐style processing approach is particularly suitable for time‐modulated encoding strategies^[^ ^187^ ^]^ and scenarios involving a substantial quantity of encoding.

#### 3.1.2 Regularization Techniques

Compared to the least squares algorithm, regularization techniques have shown significant improvement in noise tolerance, which is associated with their constraining effect on the results.^[^ ^204^, ^205^ ^]^ So far, three common regularization techniques have been extensively employed, as listed in Table 2. The first one is the *l_1_* regularization, often referred to as Lasso regression, which adds an *l_1_* norm penalty term for the target variable to the least squares model.^[^ ^21^, ^63^, ^71^, ^93^, ^99^, ^127^ ^]^ As the penalty term involves absolute values, the optimization objective is not continuously differentiable, leading to the frequent use of hard threshold iterations during the iterative process. Similar to the use of the *l_1_* norm, Tikhonov regularization, also known as ridge regression, adds an *l_2_* norm penalty term for the target variable to the least squares model.^[^ ^21^, ^27^, ^28^, ^73^, ^108^, ^119^, ^154^, ^155^, ^160^, ^164^, ^166^, ^167^ ^]^ The objective function, in this case, is continuously differentiable, thereby further reducing the computational complexity of Tikhonov regularization. In contrast to the above two regularization techniques that directly impose norm regularization on the target values to form penalty terms, total variation (TV) regularization utilizes the *l_2_* norm of the first derivative of the target values as the penalty term, which is based on the assumption that the incident spectrum is smooth, thus achieving noise tolerance objectives.^[^ ^45^, ^74^, ^79^, ^146^, ^152^ ^]^

In regularization algorithms, the selection of the regularization term coefficient is equally crucial to the form of the regularization term. Currently, the main methods for choosing the penalty term include the l‐curve criterion and generalized cross‐validation (GCV). However, both methods have limitations: the coefficients obtained by the l‐curve are not optimal; when the GCV function is monotonically decreasing, only boundary values can be obtained, making it impossible to acquire the optimal parameters.

#### 3.1.3 Other Traditional Algorithms

As summarized in Table 2, several other algorithms have also achieved commendable reconstruction results. For instance, the accelerate proximal gradient algorithm exhibits rapid convergence.^[^ ^108^, ^110^ ^]^ Heuristic algorithms, such as greedy algorithms and simulated annealing, accomplish problem‐solving with relatively less computational resource utilization.^[^ ^54^, ^96^, ^133^, ^142^ ^]^ Pre‐filtering algorithms, primarily through means like wiener filtering, enhance noise tolerance by preprocessing acquired data.^[^ ^57^, ^132^, ^134^ ^]^ There are also algorithms such as dictionary learning that have achieved good reconstruction results. Meanwhile, existing algorithms are improved to attain superior reconstruction outcomes. For example, Bian and Zhong et al. introduced an augmented LaGrangian term to the original TV regularization model, iteratively addressing challenges associated with the selection of regularization coefficients.^[^ ^79^ ^]^ Besides, combining multiple tricks to polish the performance of the algorithm is becoming common.^[^ ^72^, ^83^, ^122^, ^145^, ^190^ ^]^ For instance, Mei et al. Mei et al. proposed a bilevel optimization algorithm for a self‐adaptive spectrometer.^[^ ^122^ ^]^ This algorithm operates by encoding the spectrum into data for analysis while also providing a coarse self‐reference spectrum. The optimization occurs across both the spectrum parameter space and the algorithm parameter space, enabling a higher‐dimensional search for the global minimum cost function. This approach ensures accurate and stable spectrum reconstruction by automatically selecting the most optimal parameters.

In summary, traditional reconstruction algorithms, rooted in optimization and compressive sensing theories, have undergone extensive and sustained development, and have achieved reconstruction results at satisfactory levels. However, enhancing spectral resolution without reducing the operational wavelength range poses a challenging task for these algorithms. Fortunately, the evolution of deep learning has introduced new possibilities in this regard.

### 3.2 Deep Learning Algorithms

Deep learning, as a branch of machine learning, has its roots traceable back to the 1980s. During the nascent stages, the applications of deep learning were limited due to constrained computational resources and limited datasets. As the significant improvements in the availability of large‐scale datasets and computational power in the last decade, deep learning experienced rapid advancement. The resurgence of deep learning has been notably characterized by the introduction and refinement of sophisticated models, among which convolutional neural networks (CNN)^[^ ^206^ ^]^ and the transformer^[^ ^207^ ^]^ architectures stand out prominently. These models, with their intricate architectures and sophisticated learning mechanisms, have become pivotal in addressing complex tasks spanning diverse domains. In the past decade, deep learning has revolutionized various interdisciplinary fields, achieving remarkable success in many areas, especially image classification.^[^ ^206^, ^208^ ^]^ The advances in image classification techniques have laid the groundwork for more complex applications like spectral imaging,^[^ ^209^, ^210^ ^]^ where similar neural network architectures are employed to analyze multi‐dimensional data and reconstruct detailed spectral information.

**Figure 10:**
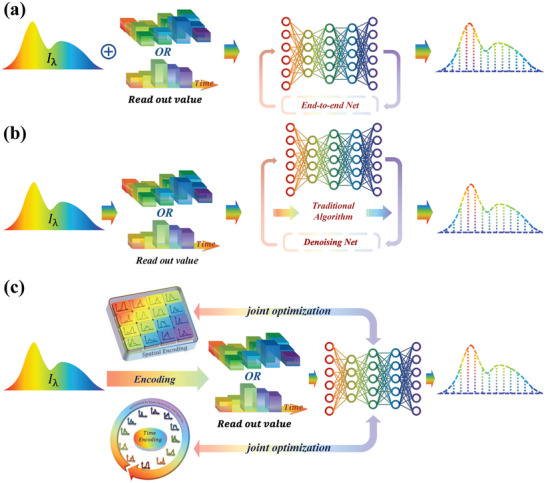
Procedures of three types of reconstruction strategies based on deep learning. a) End‐to‐end direct reconstruction networks. b) Denoising networks. c) Reverse joint optimization networks.

Deep learning has already found applications in the field of computational spectrometers, and it is anticipated that these algorithms will give rise to high spectral resolutions and improved reconstruction accuracy. Additionally, owing to the capability of providing results without the iterative process, there is a reasonable expectation that deep learning algorithms will contribute to advancements in computational speed, especially in parallel processing of multiple signals. As illustrated in **Figure** **10**, based on the application methods and roles within the reconstruction procedure, we classify deep‐learning‐based reconstruction algorithms into three categories: end‐to‐end direct reconstruction networks, denoising networks, and reverse joint design optimization networks.

#### 3.2.1 End‐to‐End Direct Reconstruction Networks

As the most common type of deep learning network, end‐to‐end direct reconstruction networks take the detector's readout signal as input and produce the reconstructed signal as output, which is depicted in Figure 10a. Typically, deep neural networks networks consist of a plain net or connection layer containing encoding information that links the input signals to subsequent layers. Following several layers of networks for data fitting calculations, the net directly outputs the reconstructed spectrum with no need for information on the encoding structure.^[^ ^31^, ^80^, ^95^, ^100^, ^116^, ^126^, ^135^, ^188^, ^194^ ^]^

It is noteworthy that the most significant difference between end‐to‐end structured deep learning networks and other reconstruction algorithms lies in their ability to train directly without the pre‐calibration process, and instead, being directly driven by data of spectral input and signals detected.^[^ ^30^, ^70^, ^112^, ^131^, ^149^ ^]^ This mode is particularly meaningful when the detector's spectral encoding strategy is challenging to quantify accurately or when there is a vast number of encodings. For instance, Ma et al. developed the MobileViT model for a micro‐nano fiber optic spectrometer.^[^ ^30^ ^]^ The training network, based on the Transformer architecture, establishes a direct connection between spectral information and detection images without the collection of feature speckle patterns for each wavelength, which substantially reduces engineering complexity.

In the context of end‐to‐end networks, key aspects include the collection of high‐quality training datasets, the architectural design of the network, and the selection of a loss function. In the context of computational spectral reconstruction, obtaining a well‐designed training dataset stands out as a serious challenge, primarily involving the utilization of detected data and spectra obtained from commercial spectrometers. This strategy may require a substantial amount of effort, as exemplified by Gan et al., who meticulously captured 10000 images for training within the 600–650 nm range.^[^ ^70^ ^]^ This approach necessitates a significant amount of real‐world data to train the model and ensure its performance and accuracy. Addressing the challenge of efficiently obtaining datasets tailored to the detector structure demands further scrutiny and exploration by researchers.

#### 3.2.2 Denoising Networks

End‐to‐end direct reconstruction networks typically employ a pre‐trained network to accomplish spectral reconstruction in a single step, whereas denoising networks rely on traditional algorithms to complete the entire computational reconstruction process. As illustrated in Figure 10b, denoising networks are usually used before conventional reconstruction algorithms, achieving noise suppression on the signal measurement. For instance, Bao et al. designed a denoising network based on a denoising autoencoder prior to the operation of the *l*_1_ regularization‐based least squares method.^[^ ^76^ ^]^ As a result, this approach still recovers the essential details of spectra even at a signal‐to‐noise ratio (SNR) of 30 dB. Even in the presence of a SNR of 30 dB, this approach accurately captured the essential details of the spectrum before the implementation of traditional reconstruction algorithms. However, since denoising networks still require spectral reconstruction using traditional algorithms, they still require a complete pre‐calibration process compared to end‐to‐end networks.

The advantage of denoising networks lies mainly in their relative universality, i.e., when the number of output channels is the same, especially for coding strategies that use CMOS/CCD cameras for signal detection, the same trained denoising network can be deployed directly without secondary training. A case in point is the application of the plug‐and‐play framework, originally designed for solving inverse problems, which has been effectively utilized in spectral image reconstruction.^[^ ^67^ ^]^

#### 3.2.3 Reverse Joint Design Optimization Networks

In traditional encoding strategies, a strategy to reduce the number of detected signals while preserving encoding performance involves obtaining as many signals as possible and subsequently screening out structures with low correlation in their output results. A more advanced approach employs numerical simulations to acquire ideal parameters for encoding systems, optimizing the encoding performance, and minimizing the number of detected signals.^[^ ^136^, ^142^ ^]^ However, as the factors involved in the encoding structure are boosted, the practical difficulty and workload associated with conventional numerical simulation methods significantly increase. In contrast, as indicated in Figure 10c, deep learning, with its ability to simultaneously consider multiple factors in parallel, is more suitable for the inverse design of complex encoding structures.^[^ ^211^, ^212^ ^]^

The current strategy employing deep learning for inverse design to optimize encoding consists of two steps: initially utilizing a network to explore the high‐diversity signals generated by an ideal encoding structure, followed by using a reverse design network to guide the practical encoding system fabrication. For example, Hao and Peng et al. proposed a network architecture, spectral encoder and decoder (SED), to achieve the optimal spectral response by extracting the weights of a single fully connected encoder.^[^ ^98^ ^]^ Subsequently, the obtained ideal spectral response is input into a reverse design network to obtain the manufacturing parameters for the corresponding filters.

## 4 Discussion

### 4.1 Cooperating Coding Structure with Algorithms

The performance ceiling of a miniaturized computational spectrometer is determined by both the quality of spectral coding and the reconstruction capability of the algorithm.

Effective spectral coding should maximize coding efficiency and robustness. Before conducting spectral measurements, appropriate metrics must be selected to evaluate these aspects. For coding efficiency, the RIC criterion from compressed sensing theory is ideal, but its NP‐hard nature makes the correlation coefficient a more practical choice. For coding robustness, the closer the coding units are to orthogonality in Hilbert space, the better the robustness. This can be assessed using the condition number; a lower condition number indicates less pathological behavior and greater stability. Overall, low correlation coefficients and small condition numbers of the coding matrix signify higher efficiency and better robustness.

Algorithm ability significantly impacts spectral reconstruction quality. High‐performance algorithms will enhance reconstruction accuracy, but they should also align with the signal characteristics of the coding structure and the exploitation stage. For instance, in scattering‐based spectral coding, where the scattering pattern is 2D, using CNN is more efficient for information extraction than traditional algorithms. Similarly, when dealing with nonlinear coded data, traditional iterative algorithms may fail to take effect, making deep learning essential for accurate reconstruction. For preliminary verification of design functionality, iterative algorithms can still be employed with lower computational requirements. However, when coding structures can be manufactured with high consistency, deep learning offers additional advantages for its one‐time training and multiple deployment features, along with optimized performance and efficiency.

### 4.2 Figure of Merits for Computational Spectrometer

In current research, figures of merits for evaluating computational spectrometers typically include the spectral detection range and spectral resolution. Some studies also measure the FWHM to evaluate the spectral resolution performance. These metrics are typical parameters for all kinds of spectrometers, but they are inadequate to comprehensively evaluate the performance of computational spectrometers. Therefore, other specific metrics for the evaluation of spectrometers employing the novel approach of computational reconstruction are needed, such as encoding efficiency and reconstruction efficiency, etc.

Encoding efficiency: In terms of encoding efficiency, a specialized performance metric might be needed for computational spectrometers:

(4) $$P_{encoding}=\frac{Operational\;range}{N_{signals}\times Spectral\;resolution}$$

Here, *N_signals_* is *n* in Equation 1, indicating the number of signals detected or encoding states during one spectral detection. This metric offers a comprehensive evaluation by considering the operational wavelength range, the number of detected signals, and the average spectral resolution. The detected signals are the signals obtained directly from a single spectral probe of the spectrometer, which is used for subsequent reconstruction using algorithms. This holistic consideration is imperative because expanding the operational wavelength range inevitably requires balancing a high average spectral resolution with a reduced number of spectral encoding points utilized. Therefore, a nuanced balance among these three factors provides a more objective and insightful measure of encoding efficiency. For example, the NIR QD spectrometer manufactured by Bian and Zhong et al. displayed the resolution improvement with the increase of QDs.^[^ ^74^ ^]^ However, once calculated by Equation 4, *P*_encoding_ exhibits a trend of a very slow decline followed by a relatively sharp decline. The value of *P_encoding_* is generally stable, proving its ability to evaluate the efficiency of the spectrometer ignoring encoder counts.

Reconstruction efficiency: To assess the algorithmic reconstruction efficiency, consideration must be given to resource utilization, including computation time and resource consumption. For traditional algorithms, as the encoding scale increases, the corresponding increase in resource consumption leads to a proportional elongation of computation time. Hence, computation time serves as a direct indicator of algorithmic resource utilization. In contrast to traditional algorithms, deep learning necessitates substantial labeled data for training and employs significant computational resources during the training process. However, upon completion of training, the direct reconstruction through a single‐layer computation, without parameter adjustments as in traditional algorithms, results in more advantageous computation resource usage and reconstruction times. Therefore, computation time might be a suitable metric to evaluate reconstruction efficiency, which is applicable to both the traditional algorithms and the deep learning method. For instance, Bian et al. introduced a meta‐attention network that significantly reduces spectral reconstruction time compared to traditional algorithms.^[^ ^100^ ^]^ Although the network achieves improved fidelity, it requires slightly more time than other deep learning methods, suggesting that reconstruction performance should also considered when talking about reconstruction efficiency.

Spectral fidelity and noise tolerance: In assessing spectral reconstruction performance, metrics such as spectral resolution or the minimum restored FWHM do not comprehensively characterize the performance of computational spectrometers. This is because when the measured signal exhibits strong sparsity, especially in the case of a single‐peaked spectrum, the reconstruction is less difficult for compressive sensing‐based computational spectrometers, compared to wide‐spectrum reconstruction. Therefore, metrics addressing wide‐spectrum reconstruction should be considered, such as mean squared error,^[^ ^190^ ^]^ RMSE,^[^ ^59^ ^]^ or fidelity,^[^ ^90^ ^]^ which are common in many works.^[^ ^25^, ^31^, ^102^, ^192^ ^]^

The noise tolerance of the spectrometer is another crucial consideration.^[^ ^213^ ^]^ In practical scenarios, micro‐vibrations, temperature variations, or other unpredictable conditions may significantly impact spectral reconstruction. Fortunately, in many cases, noise follows a Gaussian distribution, such as white noise. Therefore, evaluating noise robustness through digital domain noise simulation remains a valuable assessment method, such as using metrics like minimum SNR for noise tolerance performance. For example, the spectrometer developed by Zhao et al. demonstrates the ability to reconstruct spectral information even under conditions with 5% random noise, highlighting the robustness and resilience of their design against external perturbations.^[^ ^24^ ^]^

### 4.3 Considerations in Operating Computational Spectrometers

As one of the most promising routes to spectrometer miniaturization, computational spectrometers display many exciting advantages, including small size, low cost, relatively low system complexity, and high performance. However, there are some considerations that should be paid attention to for optimizing the capability of computational spectrometers.

1. Spike effect: According to the principle of computational spectrometers, every signal from each unit in the detector array or from each state of the tunable detectors (filters) should be involved in the reconstruction process. If there is a strong peak in the incident spectrum, some detecting units will be saturated, resulting in errors in the whole reconstructed spectrum. The impact of this situation is limited in traditional spectrometers due to their nature of light splitting, i.e., saturation only occurs at the strong peak while other spectral regions will not be affected. Therefore, it is advisable to choose light sources with suitable intensity and/or increase the linear dynamic ranges of the detectors (image sensors) for computational spectrometers.
2. Spatial uniformity of the incident light: For space‐modulated encoding strategy, it is a basic requirement that the incident light is spatially uniform and covers the entire detecting area, ensuring every unit encodes the same incident light. Otherwise, there will be a destructive impact on the reconstructed results. Appropriate optical design or reducing the area of each encoding unit is sufficient to address this issue.
3. Pre‐calibration (training): Due to the nonideal reproducibility of fabricating the encoding structures and the detectors, the pre‐calibration or training results for a given spectrometer cannot be applicable to other counterparts. Any tiny deviation in the components will lead to a substantial impact on the reconstruction results. Therefore, every computational spectrometer needs to be pre‐calibrated or trained. For scale‐up production, dedicated and automatic apparatuses might be needed for the pre‐calibration and training process.

### 4.4 Directions for Improvement of Computational Spectrometers

**Figure 11:**
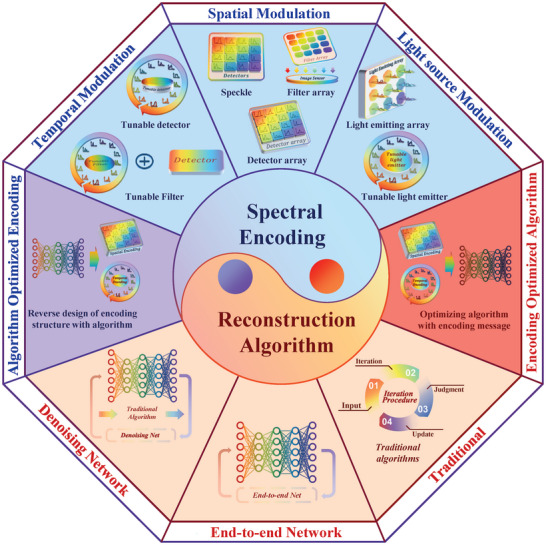
Overview of miniaturized computational spectrometers.

As shown in **Figure** **11**, currently, the development of computational spectrometers mainly encompasses the exploration of encoding structures and the optimization of reconstruction algorithms. Undoubtedly, the improvement of both aspects can enhance the overall performance of the spectrometer, but this approach of separating encoding from reconstruction for individual optimization has gradually reached a bottleneck. Therefore, an increasing number of researchers are beginning to pursue the collaborative optimization of encoding and algorithms. As illustrated in Figure 11, this primarily includes two pathways: algorithm‐optimized encoding and encoding‐optimized algorithms.

For algorithm‐optimized encoding techniques, algorithms are used to guide the design of encoding structures. Specifically, researchers simulate the spectral response and optimize the parameters and structures of the detectors used for coding based on criteria such as the autocorrelation coefficient, in order to reduce the number of detectors without sacrificing performance. For instance, Pan et al. used a multi‐point self‐coupled waveguide for spectral encoding.^[^ ^214^ ^]^ By simulating spectral responses under various parameters, they obtained encoding structural parameters with low autocorrelation coefficients, achieving a spectral resolution of 0.1 nm over a 100 nm bandwidth with 64 encoding channels. Similarly, Yang and Shen et al. applied the parameter‐constrained spectral encoder and decoder algorithm to adjust the thickness of their thin films, thereby achieving low correlation in encoded data.^[^ ^126^ ^]^

In encoding‐optimized algorithm techniques, the encoding characters are used to guide the optimization of algorithms. In this case, the algorithm is influenced not only by the spectral encoded data but also has a closer connection with the encoding structure. For instance, in the work of Dun et al., a transfer matrix method (TMM) was introduced into the end‐to‐end network design.^[^ ^117^ ^]^ The structure of the network includes not only the spectral encoded data but also the structural information of the detectors and the incident angle information. This significantly improved the performance of their network and spectrometer, making it considerably less sensitive to angles compared to typical FP cavity arrays and metasurface arrays.

In addition to the effectiveness of collaborative optimization, deep learning has brought forth new possibilities for computational spectrometers. As discussed in,^[^ ^215^ ^]^ as long as additional light field information is considered during the training process, computational spectrometers can reconstruct corresponding information. For example, Tua et al. proposed a plasmonic rainbow chip‐based spectrometer. With the assistance of deep learning, this work was able to accurately achieve polarimetric analyses and perform the detection and quantification of chiral substances.^[^ ^70^ ^]^

Moreover, following this line of thinking, material classification‐related data can be incorporated into the training network. This allows the obtained algorithm to inherently possess the capability to recognize and analyze specific scenes, yielding output results directly associated with the identified materials. For instance, Kenneth and Meng et al. realized a gas sensor with a metasurface filter array integrated with a commercial IR camera, which employs deep learning to directly get gas composition and concentration, skipping the reconstruction of their spectra.^[^ ^216^ ^]^ Achievements of such “more than spectrometry” can further broaden the applications of computational spectrometers without significant sacrifices or alterations in the original encoding strategies.

### 4.5 From Miniatured Computational Spectrometers to Hyperspectral Imaging

Traditional hyperspectral imaging techniques mainly rely on spatial scanning or wavelength scanning and are characterized by large system volumes and slow image acquisition speeds. Recently, computational spectral imaging has significantly changed the traditional routes by incorporating computation. The implementation methods mainly include amplitude encoding via coded apertures, phase encoding, and the reverse derivation of spectral information from RGB images.^[^ ^48^, ^217^, ^218^, ^219^ ^]^

With the rapid development of miniaturized computational spectrometers, they provide an alternative strategy for spectral imaging. The computational spectrometers can be assembled in an array with each spectrometer acting as a spectral pixel to collect the data cube for hyperspectral imaging. For example, Ishikawa et al. have achieved snapshot hyperspectral imaging through the implementation of 64 FP cavity arrays,^[^ ^120^ ^]^ and their integration of deep learning techniques demonstrates operational efficiency at a noteworthy full resolution of 34.4 frames per second. In 2023, Xiao, Yu, Kivshar, and Song et al. demonstrated a 10 × 10 scale hyperspectral imaging chip utilizing liquid crystal metasurfaces. This compact chip, with a footprint of only 50 × 50 µm^2^, achieves a spectral resolution of 0.4 nm and can distinguish angle‐dependent spectra, thanks to the integration of deep learning.^[^ ^95^ ^]^

Given the many similarities between computational spectral imaging and miniature computational spectrometers, such as in terms of time and spatial modulation, it's simple to associate proven technologies from computational spectral imaging with miniature computational spectrometers, as this provides a reliable route to achieve hyperspectral imaging using a single spectral detection unit. For instance, Sun, Gao, et al. achieved single‐detector hyperspectral imaging based on DMD with quantum dots, attaining a spectral resolution of 8.59 nm and an imaging scale of 128 × 128 within a 1050–1630 nm bandwidth, though the process is time‐intensive.^[^ ^220^ ^]^ More recently, Zheng, Li, et al. developed a hyperspectral imaging system by integrating tunable liquid crystals with diffractive lenses, achieving 96.3% spectral fidelity at a 500 × 500 pixel scale within the 550–700 nm range.^[^ ^179^ ^]^ Notably, this system is highly compact, with a size of only 2.12 mm × 2.12 mm, offering a promising approach for miniaturized hyperspectral imaging.

## 5 Conclusion and Outlook

In this review, we offer a comprehensive overview of computational spectrometers. Starting from the operational framework and development history, two crucial components of computational spectrometers, spectral encoding structures, and reconstruction algorithms, are summarized and analyzed in detail respectively. The advances in spectral encoding structures are presented in light of space‐modulated, time‐modulated, and light‐source spectral encoding, while the reconstruction algorithms are reviewed according to traditional and deep learning algorithms. Cooperations between the two components are considered, aiming to match the most compatible algorithm with the encoding structure. As a unique type of spectrometer, some parameters were ignored when evaluating the performance, thus figures of merits are listed and analyzed, facilitating accurate comparison between different computational spectrometers. Optimizing strategies for the computational spectrometers are summarized, pointing out that collaborative optimizing spectral encoding and reconstruction algorithms are preferred, and deep learning will be a powerful tool. Considerations that should be paid attention to when operating computational spectrometers are proposed. Additionally, extending the application of computational spectroscopy from spectrometer to hyperspectral imaging is reviewed.

Computational spectrometers simplify and reduce the sizes of the optical components at the price of introducing computational resources, which breaks the compromise between footprint and performance in traditional spectrometers and is a promising strategy for miniaturizing spectrometers. Thus, these new types of spectrometers can be used as general spectrometers or integrated with a variety of systems for widespread applications of spectral sensing.

## Conflict of Interest

The authors declare that they have no conflict of interest.
